# Temperatures and blood counts in pediatric patients treated with chemotherapy for cancer, NCT01683370

**DOI:** 10.1038/s41597-019-0112-8

**Published:** 2019-07-03

**Authors:** Luana Lavieri, Christa Koenig, Oliver Teuffel, Philipp Agyeman, Roland A. Ammann

**Affiliations:** 1 Division of Pediatric Hematology/Oncology, Department of Pediatrics, Inselspital, Bern University Hospital, University of Bern, Bern, Switzerland; 2 Division of Oncology, Medical Services of the Statutory Health Insurance Baden-Württemberg, Tübingen, Germany; 3 Department of Pediatrics, Inselspital, Bern University Hospital, University of Bern, Bern, Switzerland

**Keywords:** Paediatric cancer, Fever

## Abstract

Fever in neutropenia (FN) is the most frequent potentially lethal complication of chemotherapy in patients with cancer. The temperature limit defining fever (TLDF) for FN is based on scarce evidence. This prospective, single center observational study recruited non-selected pediatric patients diagnosed with cancer between ≥1 and ≤17 years in 2012 and 2013. Of 40 patients potentially eligible, 39 participated. Data of 8896 temperature measurements and 1873 complete blood counts (CBCs) were recorded over 289 months (24.1 years) of chemotherapy exposure time. During this time 43 FN episodes were diagnosed. In 32 episodes, FN diagnosis was based on reaching the local (i.e. Bern, Switzerland) standard TLDF of 39.0 °C; another 11 episodes had been captured by clinical judgement (i.e. temperature < 39.0 °C). These data can be used to simulate the effects of various TLDFs on the rate of FN diagnosis. We assume merging these data with other data sets is feasible.

## Abstract (summary)

**Table:** 

Design Type(s)	observation design • cohort study design • disease detection/diagnosis objective
Measurement Type(s)	body temperature • complete blood cell count
Technology Type(s)	thermometry • blood analyzer
Factor Type(s)	
Sample Characteristic(s)	Homo sapiens • whole body • Switzerland

Machine-accessible metadata file describing the reported data (ISA-Tab format)

## Background & Summary

Fever in chemotherapy-induced neutropenia (FN) is the most frequent potentially lethal complication of chemotherapy in pediatric and adult patients with cancer and should be managed as a medical emergency. Severe neutropenia is usually defined and applied in pediatric oncology practice as an absolute neutrophil count (ANC) ≤ 0.5 G/L, or ≤1.0 G/L and expected to rapidly decline^1,2^.

To reduce the risk of developing FN during chemotherapy, several prophylactic strategies have been used, such as isolation of the patient, antibiotic administration, and administration of granulocyte stimulating growth factors^3^. The current standard therapy for FN implies emergency hospitalization, empirical administration of intravenous broad-spectrum antimicrobial therapy, and antipyretics, with or without escalation to include antifungal therapy^1,4–6^.

Surprisingly, the temperature limit defining fever (TLDF), which directly influences the definition and diagnosis and thus the incidence of FN, varies considerably between different pediatric oncology centers^1,5^. This reflects the fact that an internationally accepted evidence-based TLDF definition is still missing in pediatric oncology^7^, and has been declared to be a research gap in recent pediatric FN guidelines^4^. This TLDF has direct implications on individual patient management, health-related quality of life, resource utilization, cost, and potentially treatment-related mortality^8^. Of course, efficacy – avoiding non-necessary FN diagnoses - must be weighed against safety – avoiding delays in FN diagnosis and thus empirical antibiotic therapy- for the determination of a clinically used TLDF.

The data described here^9^ had been collected during a prospective observational study (August 11, 2012 to May 31, 2013) in pediatric patients diagnosed with cancer between ≥1 to ≤17 years and treated with chemotherapy in a single center (Bern, Switzerland) applying a standard TLDF of 39.0 °C ear temperature (NCT01683370). The treating physician was free to diagnose FN below this TLDF if clinically indicated. The study was powered to determine the rate of FN episodes additionally diagnosed by lower versus standard TLDFs. Analytical results on the influence of virtually applying different lower TLDFs on FN diagnosis have been published^8^. We publish these data so they can be merged with other data, with the specific aim to generate evidence upon which the choice of a TLDF for FN diagnosis can be based. It is important to note that during the NCT01683370 study, temperature measurements after FN diagnosis, i.e., during FN, were collected too. To reflect this different clinical situation, the respective analytical results have been published separately^10^, and those data will be described separately as well.

In total, 39 of 40 potentially eligible patients participated in this study, while the parents of one patient denied informed consent. During 8799 days (289 months, 24.1 years) of chemotherapy exposure time (CET) (median CET, 199 days per patient; range, 63 to 366), 8896 temperature measurements were recorded (median rate, 26 measurements per patient per month; IQR, 8 to 53; range, 0 to 237). The median temperature measured was 37.1 °C (IQR, 36.7 to 37.6; range, 35.0 to 41.2), and 283 (3.2%) temperatures were ≥39.0 °C. In total, 43 FN episodes were diagnosed in 20 of the 39 patients. Of these, 32 FN episodes were diagnosed at temperatures ≥39.0 °C. 11 FN episodes, all with an ANC ≤ 0.5 G/L, were diagnosed at temperatures below the standard TLDF of 39.0 °C (range, 38.0 °C to 38.9 °C) for different clinical reasons. Twice, FN was not diagnosed and the patients were not hospitalized despite fever ≥39.0 °C during neutropenia. Both patients were in acute lymphoblastic leukemia maintenance therapy, had been diagnosed with an upper airway infection with good general condition within 24 hours before fulfilling FN criteria, later received oral antibiotics, with uneventful clinical course.

During the study period, 1873 CBCs were recorded (median rate of 6 CBCs per patient per month; IQR, 4 to 8; range, 2 to 23). The ANC was ≤0.5 G/L in 435 CBCs (23%). In 244 CBCs (13%), it was in-between 0.5 and 1.0 G/L. In 1032 CBCs the ANC reached >1.0 G/L and in 162 (9%) it was unknown^8^.

These data can be used to simulate the effects of various TLDFs on the rate of FN diagnosis. We assume merging these data with other data sets is feasible.

There is a data overlap of nearly 5 months (August 11, 2012, to December 31, 2012) with two separate data descriptors, both reporting on retrospectively collected patients under chemotherapy at risk to develop FN^11^, and on the corresponding FN episodes^12^ from January 1, 1993 to December 31, 2012. Temperature measurements and serial CBCs, except for those leading to FN diagnosis, are not included into the data of these two datasets.

## Methods

These methods are expanded versions of descriptions in our related work^8^.

### Study design

The data described here^9^ had been collected during the study “Pediatric FN Definition 2012 Bern”. This prospective, single-center observational study was open for recruitment from August 11, 2012 to May 31, 2013 at the Division of Pediatric Hematology/Oncology, Department of Pediatrics, Inselspital, Bern University Hospital, University of Bern, Switzerland. This hospital provides tertiary care for a population of roughly one million inhabitants. The Division of Pediatric Hematology/Oncology unit has an inpatient unit with 8 beds, plus a large outpatient unit. It treats around 40 newly diagnosed pediatric patients with all kind of malignancies per year. Besides, it performs myeloablative chemotherapy followed by autologous stem cell transplantation for the majority of Switzerland, covering around 6 million inhabitants.

Patient recruitment was closed on May 2013, and follow-up was closed on August 07, 2013, when the predetermined accrual goal of 32 FN episodes with fever ≥39 °C was reached. Participation in this observational study did not influence any diagnostic nor therapeutic decisions.

The study was conducted in accordance with the Declaration of Helsinki^13^. Before starting patient accrual, the protocol was approved by the Institutional Review Board (Ethikkommission der Universitätskinderkliniken Bern) and registered at www.clinicaltrials.com (NCT01683370). Analytical results of these data and of data studied after FN diagnosis have been published elsewhere^8,10^.

The data described here were not published together with the analytical results, in PLOS One, because they had to be fully anonymized before publication in order to comply with the current Swiss research legislation. The anonymization process was performed in 2018 to enable data sharing.

### Patients

To enter the study, the patients had to fulfill all inclusion criteria: age ≥1 year and ≤17 years at time of recruitment, chemotherapy treatment for ≥2 months because of a malignancy at time of recruitment, and written informed consent from patients and/or their parents. Infants younger than one year and patients without written informed consent were excluded.

Patients were excluded when informed consent was withdrawn, and when chemotherapy was completed, i.e., 2 weeks after the last dose of chemotherapy or date of ANC recovery >0.5 G/L, whichever came later. Of 40 potentially eligible patients, 39 participated in the study, while the parents of one patient denied informed consent.

### Clinical management

Patients were treated with chemotherapy, including myeloablative therapy followed by autologous peripheral blood stem cell transplantation, or multimodal therapy, according to internationally established protocols.

In inpatients, ear temperature was measured routinely twice a day. Additional measurements were made when fever was suspected, as well as before and during transfusions and medications known to potentially induce fever. In outpatients, parents were instructed to measure ear temperature when they suspected fever and to note the results on paper forms.

CBCs were routinely performed once or twice a week according to disease. Additional CBCs were performed if temperature was ≥39.0 °C, in case of reduced general condition or other problems. Patients with fever without severe neutropenia (ANC > 0.5 G/L) and in good general condition were allowed to take oral paracetamol. Patients with fever and severe neutropenia (ANC ≤ 0.5 G/L) or in reduced general condition were seen in the emergency department and usually diagnosed with FN, implying emergency hospitalization and empirical therapy with intravenous broad-spectrum antibiotics, usually ceftriaxone plus amikacin and antipyretics. Thus, the management of FN was similar to the current standard therapy described above^1,3,4,6^.

### Fever in neutropenia

During this study, FN was diagnosed when a patient had chemotherapy-induced severe neutropenia and fever (single ear temperature ≥39.0 °C) or the physician in charge decided to start empirical antimicrobial treatment based on her/his clinical judgement. With increasing or plateau temperature, this TLDF corresponds to 39.1 °C core temperature, and to 38.4 °C axillary temperature^14^.

Neutropenia was defined as a peripheral blood ANC ≤ 0.5 G/L, or ≤1 G/L and expected to decline, induced by chemotherapy, i.e. ≥7 days after first start of chemotherapy, irrespective of the setting.

The start of FN corresponded to the time of hospitalization and start of empirical antimicrobial therapy for FN, as clinically decided by the treating physician. Patients were usually discharged if they were afebrile for ≥48 hours and clinically well, initial blood cultures were negative, and leukocyte counts and/or ANC were rising (irrespective of their absolute value)^8^. The FN episode ended with the end of any antimicrobial therapy for FN. Restarting antimicrobial therapy within 7 days and with persistent neutropenia was considered to belong to the same FN episode^8^.

### Data coverage of this study

This study collected data on temperature measurements and CBCs before and after the FN episode.

Data on temperature measurements and CBCs performed during the FN episode are published elsewhere^10^ and are not part of the data described here. Data collected at FN diagnosis and at the end of the FN episode are published in both datasets.

## Data Records

A single data record resulted from this study (Table 1). It contains 39 data files, one per patient (File Pediatric_FN_Definition_2012.Patient_xxxx.csv.), where xxxx refers to a patient-specific random four digit number between 1000 and 9999, plus a key file (Pediatric_FN_Definition_2012.Key.csv). Data are available within the figshare repository^9^.

**Table 1:** Overview.

Observations	Time covered	Center	Data source	Data
Patients	08.2012–08.2013	Bern	Chart records	Pediatric_FN_Definition_2012.Patient_xxxx.csv

## Technical Validation

### Data collection

An experienced pediatric oncology nurse extracted information from the paper forms brought in by parents as described above and from patient charts within the setting of a prospective clinical study. This information was checked for plausibility and, if not plausible, for agreement with charts by a pediatric oncologist (RAA) before analysis^8^. The data was then entered into a spreadsheet.

### Reduction of recruitment bias

39 of 40 potentially eligible patients participated in the study and therefore, a relevant recruitment bias can be excluded. Correspondingly, the distribution of gender (23 males and 16 females), age at diagnosis (median, 7.4 years; range, 1.2 to 16.7) and diagnostic groups (acute lymphoblastic leukemia in 18 patients, acute myeloid leukemia in 2, Non-Hodgkin lymphoma in 2, central nervous system tumor in 5, and other solid tumors in 12) were comparable with an unselected population of pediatric patients with cancer^8^.

### Increasing reliability of information

A simple restricted definition of FN episodes based on verifiable quantitative information both on fever and on neutropenia was used in the vast majority of FN diagnoses. Additional FN episodes could be diagnosed by the treating physician before the standard TLDF if clinically indicated^8^.

Temperature was always measured in the ear by infrared tympanic thermometry using the same device, a Braun ThermoScan 5 (IRT 4520; Braun GmbH, Kronberg, Germany; steps displayed, 0.1 °C; accuracy, ±0.2 °C; clinical repeatability, ±0.14 °C), during hospitalization, in the outpatient clinics, and at home.

During febrile and non-febrile periods in children, infrared tympanic thermometry measurements more accurately reflect core temperatures than any other measurement site^14^.

### Anonymization process

Data were irreversibly anonymized before publication in order to comply with the current Swiss research legislation. First, a four digit random number, not linkable to the patient number used in the initial study, was used per patient. Second, diagnoses, age and gender, which are all classified as indirect identifiers are not apparent in the published dataset. Third, the date of study start, classified as direct identifier^15,16^, was always replaced by day 0, to prevent identification. Further dates were replaced by numbers indicating the time distance (in days) from day 0. The real time of the day is expressed as fraction of the day. Fourth, CBCs might be considered as identifying numbers^15,16^, because of the almost uniquely occurring data combination of hemoglobin, leukocytes, platelets and ANC. Different anonymization techniques, such as data aggregation, rounding, swapping data or adding noise had been discussed^17,18^. Weighing advantages and disadvantages we finally decided to use no anonymization method and to publish original data, in order not to hamper the future analysis of these published data.

## ISA-Tab metadata file

Download metadata file

## Data Availability

This study did not use any computer codes to generate the dataset. Microsoft Excel was used to enter, store and quality check the collected data.
